# Vitamin D Deficiency at Melanoma Diagnosis Is Associated with Higher Breslow Thickness

**DOI:** 10.1371/journal.pone.0126394

**Published:** 2015-05-13

**Authors:** Candy Wyatt, Robyn M. Lucas, Cameron Hurst, Michael G. Kimlin

**Affiliations:** 1 AusSun Research Laboratory, Institute of Health and Biomedical Innovation, Queensland University of Technology, Brisbane, Australia; 2 National Centre for Epidemiology and Population Health, The Australian National University, Canberra, Australia; 3 Telethon Kids Institute, University of Western Australia, Perth, Australia; 4 Faculty of Public Health, Khon Kaen University, Khon Kaen, Thailand; Medical University of Gdańsk, POLAND

## Abstract

**Background:**

Epidemiological evidence shows that people with thicker, or higher stage, melanomas have lower vitamin D status compared to those with thinner tumours. Evidence from experimental studies is inconsistent, but some suggest that administration of vitamin D metabolites can decrease tumour aggressiveness.

**Objectives:**

Determine the relationship between vitamin D status at diagnosis and melanoma thickness (as an indicator of prognosis), in a subtropical setting with high melanoma incidence.

**Methods:**

We recruited 100 melanoma patients in Brisbane, Australia within days of their diagnosis. Data on factors previously associated with melanoma risk or prognosis were collected by questionnaire and physical examination. Serum for 25-hydroxyvitamin D3 [25(OH)D] levels was collected prior to wider excision biopsy; histological indicators of prognosis were obtained from pathology reports. We used multivariable logistic regression models to analyse the association between Breslow thickness (≥0.75 mm compared to <0.75 mm), Clark level (2–5 compared to 1) and presence of mitoses, and vitamin D status.

**Results:**

Serum 25(OH)D <50 nmol/L (versus ≥50 nmol/L) was associated with a nearly four-fold increase in risk of having a thicker tumour (Adjusted OR = 3.82, 95% CI: 1.03, 14.14; *p* = 0.04, adjusted for age, sex, skin phototype, body mass index and season at diagnosis). There was no significant association with Clark level or presence of mitosis. Serum 25(OH)D levels in the highest quartile (≥69.8 nmol/L) were not associated with a more favourable prognosis.

**Conclusions:**

Vitamin D deficiency at the time of melanoma diagnosis is associated with thicker tumours that are likely to have a poorer prognosis. Ensuring vitamin D levels of 50 nmol/L or higher in this population could potentially result in 18% of melanomas having Breslow thickness of <0.75 mm rather than ≥0.75 mm.

## Introduction

Cutaneous melanoma is common in developed countries: it was the 10^th^ most common cancer in the very highly developed regions of the world in 2012 and 230,000 new cases were reported worldwide [1]. Incidence rates continue to increase in most countries with predominantly fair-skinned populations, particularly in older people [2]. Mortality rates are also increasing in many of these countries, and there were over 55,000 deaths from melanoma in 2012 [1].

Survival from melanoma is best predicted by Breslow thickness (the microscopic measurement of the distance between the upper layer of the epidermis and the deepest point of tumour spread) and the presence of ulceration [3, 4], as well as mitotic rate [5, 6]. Level of invasion (Clark level) is a significant predictor in patients with thin melanomas (1 mm or less) [4]. Survival rates also depend on the melanoma stage, with 10-year survival varying from 93% for the thinnest tumours with no evidence of spread (Stage 1A) to 39% for thick tumours with evidence of metastasis (Stage IVb) [7].

Exposure to solar ultraviolet radiation (UVR) is the major risk factor for melanoma in fair-skinned populations [3, 8–10]. However, previous research has suggested that higher (compared to lower) self- reported lifetime intermittent sun exposure is associated with a lower risk of death within five years of diagnosis (*HR* = 0.6, 95% CI = 0.3, 1.0, *p* = 0.04) [11]. One hypothesis is that these apparent beneficial effects of sun exposure on survival might be mediated through improvement in vitamin D status [12].

Experimental studies provide mixed support for this hypothesis. Cultured melanoma cells express the 25-hydroxylase enzyme that converts the circulating form of vitamin D, 25-hydroxyvitamin D_3_ (25(OH)D), to the biologically active form, 1,25-dihydroxyvitamin D_3_ [1,25(OH)_2_D_3_] [13], but recent evidence suggests that most melanoma cell lines are resistant to the anti-proliferative effects of 1,25(OH)_2_D_3_ [14]. In immunosuppressed mice, administration of pharmacological doses of 1,25(OH)_2_D_3_ suppressed melanoma growth and inhibited metastases for one human solid xenograft melanoma line, but not another [15]. Nevertheless, studies in other cancers demonstrate regulatory effects of 1,25(OH)_2_D_3_ on cellular growth and angiogenesis that would plausibly improve cancer prognosis.

There is more consistency in the findings from the small number of epidemiological studies examining this question. Serum 25(OH)D levels (at variable times post-diagnosis) were significantly lower in patients with Stage IV compared to with Stage 1 melanoma (median 33 nmol/L compared to 41 nmol/L, *p* = 0.006 [16]; 21 nmol/L compared to 36 nmol/L, *p* = 0.04 [17]) in two studies. In another study there was a significant trend toward lower 25(OH)D levels (taken 3–6 months after diagnosis) in association with higher Breslow thickness (adjusted mean 25(OH)D: <0.75 mm = 56 nmol/L; >3 mm = 49 nmol/L) [18]. A major issue with each of these studies is that blood sampling occurred at various times after the diagnosis of melanoma and could reflect the increased sun awareness and protection that occurs after skin cancer diagnosis.

Australia has the highest incidence rate of cutaneous melanoma in the world [1, 19] due to high levels of UVR and an outdoors lifestyle. Yet even in this sunny environment 23% of the population had serum 25(OH)D levels of <50 nmol/L (commonly considered to be sub-optimal) in the most recent Australian Health Survey [20]. A causal link between vitamin D deficiency at diagnosis, and poorer prognosis after diagnosis, is of considerable public health importance, amenable to intervention, particularly where melanoma incidence is high.

We examined serum 25(OH)D levels and sun exposure history at melanoma diagnosis in relation to histopathological indicators of prognosis: Breslow thickness [21], ulceration [3], Clark level [22], and presence of mitotic activity [5, 6] in patients with newly diagnosed melanoma in Brisbane, Australia.

## Materials and Methods

### Study population and setting

Participants were patients aged 18 years and older with histologically confirmed cutaneous melanoma, recruited prior to wider-excision surgery from two plastic surgery practices in Brisbane, Australia. Brisbane is geographically situated at Latitude 27.5° South, Longitude 153° East. Recruitment took place between 1 July 2010 and 27 July 2011. Exclusion criteria included: diagnosis made from metastatic or lymph node tissue; self-reported pre-existing conditions that could interfere with the vitamin D pathway such as chronic liver or kidney disease; transplant recipients; or receiving high dose calcium therapy.

The Human Research Ethics Committee of the Queensland University of Technology (QUT) approved the conduct of the QUT Melanoma Study, and written informed consent was obtained from all participants (QUT HREC Approval number: 0900000681).

### Study procedures

In this region where the incidence of melanoma is high, when a melanoma is diagnosed by biopsy or shave excision patients are usually “fast-tracked” to consult a surgeon within days of diagnosis, with wider-excision surgery scheduled as soon after that as is possible.

Participants were recruited prior to wider-excision surgery and provided with a questionnaire to be completed and returned at the post-operative visit. Questionnaire data included demographics (age, sex, birthplace, highest level of education completed, occupational history), skin phenotypic characteristics as a teenager (natural hair colour and mole and freckle density were ascertained by matching answers with pictures depicting different categories), burning and tanning propensity, past history of sun exposure over the life course (obtained by asking place of residence and time spent in the sun on work days and non-work days), historical use of sun protection (sunscreen, hats, sunglasses, long sleeves, shade), history of sunburns resulting in either blistering, prolonged soreness or peeling of the skin, and history of previous melanoma.

At the post-operative appointment a registered nurse trained in skin examination (CW) undertook a count of naevi that were 2 mm or greater in diameter on the head, neck, arms and trunk (from the hipline up), and the freckle and solar lentigo density (none, few/some, heavy) in three regions: the forearms, the face and the shoulders. Natural skin pigmentation (left upper, inner arm) was measured using reflectance spectrophotometry (Minolta CM2500d spectrophotometer, Minolta Camera Company, Ltd., Osaka, Japan). Data were also collected on use of vitamin supplements, height and weight.

Data relating to melanoma type, anatomic site, presence of solar elastosis and the histopathological prognostic indicators [3, 5, 6] of Breslow thickness, ulceration, Clark level and presence of mitotic activity, were obtained from the histopathology reports of both the biopsy and wider-excision samples.

A blood sample was collected prior to wider excision surgery; serum was aliquoted and stored at -80°Celsius until the completion of the study. The 25(OH)D concentration was measured in two batches using a commercial chemiluminescent immunoassay (LIAISON 25_OH Vitamin D TOTAL assay: DiaSorin, Inc., Stillwater, MN), with intra-assay variability of 3–6% and inter-assay variability of 6–9%. The laboratory is certified by the Vitamin D External Quality Assessment Scheme (DEQAS).

### Statistical analysis

Data were described using means and standard deviations, medians and interquartile ranges or numbers and proportions, depending on the data type and the distribution. Relationships between variables were tested in univariate analyses using Chi-squared or Fisher’s exact tests for categorical variables, and Kruskal-Wallis, Anova or Students t-test for comparisons of continuous variables across categories of independent variables.

Serum 25(OH)D level was treated as a continuous variable and also dichotomised to examine the effect of low vitamin D status using both the lowest quartile (less than 45.3 nmol/L) and a commonly used cut-point: less than 50 nmol/L. The latter is commonly considered to be sub-optimal [23, 24]. We also investigated whether higher vitamin D status (highest quartile: ≥69.8 nmol/L) was associated with histological indicators of melanoma prognosis.

Natural skin phototype was derived from the L* and b* spectrophotometric measurements using the formula: [arctangent((L*-50)/ b*)]*180/3.14159 to calculate the ITA (Individual Topography Angle) value [25]. ITA was treated as both a continuous variable (ITA value) and as an ordinal categorical variable representing the three ITA skin types seen in the study: intermediate/medium (>28° to ≤41°), light/fair (>41° to ≤55°) and very light/very fair (>55° to ≤90°).

BMI (Body Mass Index) was calculated by dividing weight in kilograms by height in meters squared (kg/m^2^). Supplement usage was classified as takes no supplements, takes a multivitamin, and takes vitamin D supplements. Naevus burden was grouped into 0–9 naevi, 10–39 naevi, and ≥40 naevi to reflect low, medium and high burden.

The prognostic outcome indicator of Breslow thickness was dichotomised (≥0.75 mm versus <0.75 mm) according to past evidence that this is a rational cut-point [21]. More recently the AJCC [7] has described a cut-point of 1.00 mm, but due to the small number of study melanomas greater than 1.00 mm (*n* = 12) in this study, the 0.75 mm cut-point was preferable. The other outcome indicators were also dichotomised: Clark level (levels 2–5 versus level 1), ulceration (present versus absent) and mitotic activity (present versus absent). Ultimately, ulceration was not included in multivariable analyses due to low numbers (*n* = 4). If participants had been diagnosed with more than one melanoma simultaneously, only the first melanoma reported in the histopathology for the biopsy specimen was included in the analysis. Any melanomas not reported as lentigo maligna, superficial spreading or nodular melanoma, were grouped together as “other melanoma”.

We used logistic regression to examine the relationship between vitamin D status (as both continuous and categorical variables as noted above) and each of the dichotomous outcomes. Potential confounders identified from the univariate analyses were entered into a multivariable logistic regression model and were retained in the model if their inclusion changed the coefficient of the main exposure of interest [25(OH)D] by 5% or greater. Age (in years) and sex were retained in all multivariable models. A *p* value of less than 0.05 (two-sided test) was considered statistically significant. Tests for linear trend were conducted by fitting ordered categories of a variable as a single ordinal variable in the regression models; trend *p* values were based on the Wald test. Participants with missing data were excluded from analyses using those variables. All analyses were carried out in Stata IC version 11.2 (Stata Corporation, 2009. College Station, TX).

## Results

Of the 102 patients who satisfied the inclusion and exclusion criteria, 100 participated in the study (98% participation rate). Histopathology reports were obtained for all participants, 99% of participants completed the questionnaire, 98% took part in the skin examination, and blood samples for 25(OH)D analysis were obtained from all participants prior to wider excision surgery. The youngest participant was 26 years old, and the oldest 89 years. The mean age was 61 years (*SD* = 13). Average body mass index (BMI) was 27 kg/m^2^ (*SD* = 5); 40% of participants reported ever smoking and 6% currently smoked. Vitamin D supplement use was reported by 9% of participants and 2% reported taking a multivitamin. A previous melanoma was reported by 13% of participants. In this group, 77% were 60 years or older and equally distributed by sex. The deepest reported Breslow thickness was 7.8 mm.

The mean serum 25(OH)D concentration was 58 nmol/L (*SD* = 19), with a range from 16–114 nmol/L. Over one-third of participants (36%) had serum 25(OH)D level of <50 nmol/L. Variation in serum 25(OH)D levels and vitamin D status according to baseline characteristics are shown in Table 1. Sub-optimal 25(OH)D levels were more common in those with higher body mass index (BMI) and fairer skin type, although these trends were not statistically significant. However, when Individual Topography Angle (ITA) skin type was treated as a continuous variable it was significantly associated with 25(OH)D concentration (beta coefficient = -0.66, 95% CI = -1.22, -0.11, *p* = 0.02). As expected, 25(OH)D levels <50 nmol/L were most frequent in winter and least in summer, while 25(OH)D levels in the highest quartile were most common in samples taken in autumn. A higher proportion of the group who reported a previous melanoma had serum 25(OH)D levels <50 nmol/L, but this was not statistically significant.

**Table 1:** Baseline characteristics of participants in relation to 25(OH)D concentration and vitamin D status (<50 nmol/L and upper quartile [≥69.8 nmol/L]).

Characteristic	Frequency *n* (%)	25(OH)D level (nmol/L) mean (*SD*)	25(OH)D <50 nmol/L *n* (%)	Upper quartile 25(OH)D (≥69.8 nmol/L) *n* (%)
**Age**				
<60 years	40 (40)	50.1 (17.8)	14 (35)	8 (20)
≥60 years	60 (60)	59.6 (20.1)	22 (37)	17 (28)
*p*-value		0.37	0.87	0.35
**BMI**				
<25.0	34 (35)	60.0 (18.4)	10 (29)	9 (27)
25–29.9	36 (37)	59.3 (22.1)	13 (36)	11 (31)
30+	27 (28)	53.4 (16.3)	13 (48)	4 (15)
*p* for trend		0.36	0.32	0.34
**ITA skin type**				
Intermediate/medium	8 (8)	67.5 (31.8)	2 (25)	3 (38)
Light/fair	70 (72)	58.0 (17.6)	25 (36)	17 (24)
Very light/very fair	20 (20)	53.4 (18.1)	9 (45)	4 (20)
*p* for trend		0.22	0.58	0.62
**Season at diagnosis**				
Winter	29 (29)	52.0 (17.3)	15 (52)	4 (14)
Spring	25 (25)	58.3 (22.5)	11 (44)	4 (16)
Summer	20 (20)	62.3 (19.4)	3 (15)	7 (35)
Autumn	26 (26)	61.8 (16.8)	7 (27)	10 (39)
*p* for trend		0.18	0.04	0.09
**Sunscreen use 5 years ago**				
Most of the time	7 (7)	60.0 (19.6)	3 (43)	2 (29)
Sometimes	31 (31)	61.0 (19.9)	11 (36)	8 (26)
Rarely/never	61 (62)	56.4 (19.1)	22 (36)	15 (25)
*p* for trend		0.54	0.95	1.00

Values not summing to 100 indicate missing data.

*SD*: standard deviation; BMI: body mass index; ITA: Individual topography angle.

Analyses of baseline characteristics of patients in relation to the prognostic indicators are reported in Table 2 and show that those with fairer skin type were more likely to present with thicker melanomas and for mitoses to be present. There was no association with smoking categorized as ever vs. never or as a continuous variable of total number of pack years (data not shown). There was a positive association between BMI and Breslow thickness (*OR* = 1.12, 95% CI = 1.01, 1.26, *p* = 0.04 per unit increase in BMI). Higher Breslow thickness was more commonly found in those reporting medium or high sun exposure, more than 20 childhood sunburns, and in those with presence of solar elastosis, but none of these associations was statistically significant (see Table 2). Similarly there was no association between serum 25(OH)D concentration and the anatomic location or the histological features of the melanoma in crude analyses (see Table 3).

**Table 2:** Baseline characteristics of participants in relation to Breslow thickness, Clark level and mitotic activity.

Characteristics	Frequency *n* (%)	Breslow thickness (≥0.75 mm) *n* (%)	Clark level (Levels 2–5) *n* (%)	Mitotic activity (present) *n* (%)
**Sex**				
Male	56 (56)	8 (14)	29 (52)	15 (27)
Female	44 (44)	9 (20)	26 (59)	8 (18)
*p*-value		0.42	0.47	0.31
**Age**				
<60 years	40 (40)	9 (23)	23 (58)	10 (25)
≥60 years	60 (60)	8 (13)	32 (53)	13 (22)
*p*-value		0.23	0.68	0.70
**BMI**				
<25.0	34 (35)	3 (9)	17 (50)	3 (9)
25–29.9	36 (37)	7 (19)	20 (56)	11 (31)
30+	27 (28)	6 (22)	16 (59)	8 (30)
*p* for trend		0.33	0.79	0.05
**ITA skin type**				
Intermediate/medium	8 (8)	0 (0)	3 (38)	0 (0)
Light/fair	70 (72)	9 (13)	37 (53)	13 (19)
Very light/very fair	20 (20)	7 (35)	14 (70)	9 (45)
*p* for trend		0.03	0.24	0.02
**Reported UVR exposure**				
Low	18 (19)	2 (11)	9 (50)	4 (22)
Medium	38 (41)	8 (21)	21 (55)	6 (16)
High	37 (40)	5 (14)	20 (54)	10 (27)
*p* for trend		0.62	0.93	0.52
**Naevi burden at diagnosis**				
0–9	25 (25)	3 (12)	15 (60)	5 (60)
10–39	45 (46)	8 (18)	21 (47)	12 (27)
40+	28 (29)	5 (18)	18 (64)	5 (18)
*p* for trend		0.83	0.30	0.68
**Solar elastosis** ^**a**^				
Present	34 (71)	6 (18)	20 (59)	10 (29)
Absent	14 (29)	2 (14)	8 (57)	3 (21)
*p*-value		0.57	0.92	0.73
**Childhood sunburns**				
0–5	40 (40)	6 (15)	21 (53)	7 (18)
6–20	44 (45)	7 (15)	27 (61)	12 (27)
> 20	15 (15)	3 (20)	6 (40)	3 (20)
*p* for trend		0.87	0.34	0.58
**Skin checks in last 3 years**				
3 or more	46 (47)	5 (11)	20 (44)	10 (22)
1 or 2	28 (28)	6 (21)	16 (57)	7 (25)
Nil	25 (25)	5 (20)	18 (72)	5 (20)
*p* for trend		0.41	0.07	0.90

UVR: ultraviolet radiation.

^a^Histopathology reporting for solar elastosis available for *n* = 48 melanomas

**Table 3:** Summary descriptive statistics (frequency, mean, median, standard deviation and range) of serum 25(OH)D (nmol/L) and associations with anatomic site and histological characteristics of the melanomas.

		25-Hydroxyvitamin D concentration (nmol/L)				
Characteristic (*N*)	*n* (%)	Mean	*SD*	Median	Range	*P*-value
**Anatomic site (100)**						0.87
Head & neck	27 (27)	56.8	16.6	58.6	21.1–99.6	
Trunk	37 (37	59.1	17.1	58.9	37.9–114.0	
Upper limbs	12 (12)	64.3	23.5	61.6	24.3–104.0	
Lower limbs	24 (24)	55.1	22.9	54.9	15.8–101.0	
**Melanoma type (100)**						0.55
LM^a^	29 (29)	59.9	17.7	59.8	21.1–99.6	
SSM^b^	54 (54)	57.5	20.6	58.6	15.8–114.0	
Nodular	2 (2)	73.3	43.5	73.3	42.5–104.0	
Other melanomas^c^	15 (15)	55.2	14.3	52.7	32.8–85.7	
**Breslow thickness (100)**						0.08
<0.75 mm	83 (83)	59.7	18.0	59.0	15.8–114.0	
≥0.75 mm	17 (17)	50.7	23.4	42.5	24.3–104.0	
**Clark level (100)**						0.22
Level 1	45 (45)	60.8	20.7	59.6	15.8–114.0	
Level 2–5	55 (55)	56.0	17.9	53.3	24.3–104.0	
**Mitosis (100)**						0.47
Absent	77 (77)	58.9	18.8	58.6	15.8–114.0	
Present	23 (23)	55.6	20.9	57.8	24.5–104.0	

^a^ Lentigo maligna melanoma

^b^ Superficial spreading melanoma

^c^ Includes lesions reported by histopathology report as “malignant melanoma” and other variations of malignant melanoma

In separate multivariate logistic regression models with adjustment for confounding factors (see Table 4), higher 25(OH)D levels (per 10 nmol/L) were associated with decreased odds of having an indicator of poorer prognosis, but this was not statistically significant for any outcome.

**Table 4:** Relationship of 25-hydroxyvitamin D concentration (per 10 nmol/L) to the histopathological indicators of melanoma prognosis with adjustment for confounding factors.

Model	Crude *OR*	95% CI	*P*-value	Adjusted *OR*	95% CI	*P*-value
Breslow thickness(≥0.75 mm)	0.75	0.54, 1.02	0.07	0.78^**a**^	0.55, 1.11	0.17
Clark level(Levels 2–5)	0.88	0.72, 1.09	0.26	0.93^**b**^	0.71, 1.22	0.60
Mitotic activity(present)	0.91	0.71, 1,17	0.47	0.96^**c**^	0.70, 1.33	0.82

*OR*: odds ratio; CI: confidence interval.

^a^ Adjusted for age, sex, ITA value, BMI, season at diagnosis

^b^ Adjusted for age, sex, ITA value, BMI, season at diagnosis, naevi burden at diagnosis, number of primary school sunburns

^c^ Adjusted for age, sex, ITA value, BMI, season at diagnosis, naevi burden at diagnosis, number of primary school sunburns

However, lower vitamin D status, either in absolute (<50 nmol/L) or relative (lowest quartile compared to the other three quartiles) terms, was associated with increased odds of having higher Breslow thickness (see Table 5). Indeed, there was a nearly four-fold increase in the odds of higher Breslow thickness in association with 25(OH)D <50 nmol/L and there was a greater than five-fold increase for the lowest quartile compared with the highest, where the cut-off point was a little lower (45.3 nmol/L). There was no association between vitamin D status and either Clark level (see Table 6) or presence of mitotic activity (see Table 7). In addition, a 25(OH)D level in the highest quartile (compared to the other three quartiles) was not associated with a reduced risk of having an indicator of poorer prognosis. Inclusion of past history of melanoma or use of vitamin D supplementation did not alter the effect estimates reported here.

**Table 5:** Vitamin D status (lower quartile [<45.3 nmol/L], <50 nmol/L, upper quartile [≥69.8 nmol/L]) at time of diagnosis, in relation to Breslow thickness (≥0.75 mm vs. <0.75 mm).

Vitamin D status	Crude *OR*	95% CI	*P*-value	Adjusted *OR* ^a^	95% CI	*P*-value
≥45.3 nmol/L	Reference			Reference		
<45.3 nmol/L	5.30	1.72, 16.40	0.01	5.03	1.32, 19.09	0.02
≥50 nmol/L	Reference			Reference		
<50 nmol/L	3.59	1.18, 10.93	0.03	3.82	1.03, 14.14	0.04
≥69.8 nmol/L	Reference			Reference		
<69.8 nmol/L	0.97	0.28, 3.34	0.96	0.83	0.19, 3.58	0.80

^a^ Adjusted for age, sex, ITA value, BMI, season at diagnosis

**Table 6:** Vitamin D status (lower quartile [<45.3 nmol/L], <50 nmol/L, upper quartile [≥69.8 nmol/L]) at time of diagnosis, in relation to Clark level (Levels 2–5 vs. Level 1).

Vitamin D status	Crude *OR*	95% CI	*P*-value	Adjusted *OR* ^a^	95% CI	*P*-value
≥45.3 nmol/L	Reference			Reference		
<45.3 nmol/L	1.64	0.64, 4.18	0.30	1.28	0.38, 4.33	0.69
≥50 nmol/L	Reference			Reference		
<50 nmol/L	1.77	0.76, 4.11	0.19	1.83	0.62, 5.44	0.27
≥69.8 nmol/L	Reference			Reference		
<69.8 nmol/L	2.05	0.81, 5.23	0.13	1.56	0.48, 5.12	0.46

^a^ Adjusted for age, sex, season at diagnosis, ITA value, supplements, BMI, naevi burden at diagnosis, primary school sunburns

**Table 7:** Vitamin D status (lower quartile [<45.3 nmol/L], <50 nmol/L, upper quartile [≥69.8 nmol/L]) at time of diagnosis, in relation to mitotic activity (present vs. absent).

Vitamin D status	Crude *OR*	95% CI	*P*-value	Adjusted *OR* ^a^	95% CI	*P*-value
≥45.3 nmol/L	Reference			Reference		
<45.3 nmol/L	2.47	0.89, 6.80	0.08	2.21	0.56, 8.68	0.26
≥50 nmol/L	Reference			Reference		
<50 nmol/L	1.60	0.61, 4.21	0.34	2.12	0.72, 6.19	0.17
≥69.8 nmol/L	Reference			Reference		
<69.8 nmol/L	0.40	0.27, 2.39	0.70	0.40	0.10, 1.69	0.21

^a^ Adjusted for age, sex, season at diagnosis, ITA value, BMI, reported adult sun exposure, adult sunburns, naevi burden at diagnosis, primary school sunburns

## Discussion

Here we found that vitamin D deficiency at the time of melanoma diagnosis was associated with higher Breslow thickness, a known marker of poorer prognosis. There was no significant association between vitamin D and two other histopathological indicators of prognosis. Furthermore, we found no significant association between markers of prognosis and past or recent sun exposure, history of childhood sunburns or solar elastosis.

Many studies have investigated risk factors for melanoma, but less is known about the factors that influence melanoma prognosis. A previous study showed an inverse association between risk of death from melanoma and higher levels of sun exposure prior to diagnosis of the melanoma (based on questionnaire data on intermittent sun exposure [high vs. low], severe sunburn [yes/no] and presence of solar elastosis [yes/no]) [11]. In our smaller study, at a lower latitude, no participant reported a history of no severe sunburns (compared to *n* = 173 [33% of the study sample]) in Berwick et al), and 15% reported more than 20 severe sunburns. Furthermore, we report here on markers of melanoma prognosis, rather than death. Our findings did not corroborate those of this previous study set in Connecticut, a much higher latitude location (42°N), possibly because of the much higher sun exposure experience of our participants (and the lack of a truly “low” sun exposure group). Indeed, it is important to note the difference in absolute received UVR dose of “high” and “low” sun exposure in studies from lower, compared to higher, latitudes [26].

A few recent studies have suggested that there may be independent beneficial effects of sun exposure and 25(OH)D levels for some disease outcomes [27, 28]. For melanoma, one plausible pathway for a beneficial effect of sun exposure itself on melanoma prognosis is through alteration of the skin microenvironment [29, 30]. Plausible pathways for a beneficial effect of higher 25(OH)D levels on cancer prognosis are well-described [31, 32]. We are unable to separate these effects on prognosis in this cross-sectional study. Serum 25(OH)D level may be the important factor, or could be a marker of higher recent (and possibly usual) sun exposure, with the latter the true protective factor for melanoma thickness at diagnosis. Nevertheless, here we found no significant association between the prognostic indicators and measures of past sun exposure.

Our results are consistent with previous studies from the Northern Hemisphere [16–18] that have investigated associations between vitamin D status and melanoma prognosis. Furthermore, the results reported here strengthen the causal attribution of previous findings by ruling out an explanation of reverse causality due to lowered sun exposure (and 25(OH)D levels) as a result of the diagnosis of melanoma. Similar associations between serum 25(OH)D level and prognosis have been shown for other cancers such as gastric, breast and prostate [33–35].

A causal role for vitamin D in melanoma prognosis is also supported by recent findings of disturbance of the expression of vitamin D-related genes within melanomas. Both locally and systemically, levels of the active form of vitamin D [1,25(OH)_2_D_3_] are dependent on the action of 1α-hydroxylase enzyme (encoded by *CYP27B*1) that converts 25(OH)D to 1,25(OH)_2_D_3_ and the catabolic 24-hydroxylase enzyme (encoded by *CYP24A1*) that converts 1,25(OH)_2_D_3_ to the much less biologically active 24,25-dihydroxvitamin D_3_ [24,25-(OH)_2_D_3_]. The effects of 1,25(OH)_2_D_3_ on gene transcription are mediated through binding with a nuclear vitamin D receptor (VDR). Reduced expression of *CYP27B1* in primary melanomas was associated with shorter overall survival [36] in one study, while expression of *CYP24A1* was higher in naevi and early stage melanomas compared to normal skin, but decreased with melanoma progression in another [37]. In particular, a lack of *CYP24A1* expression was associated with poorer prognosis. The findings suggest that elevated *CYP24A1* expression may be important for the formation of melanocytic naevi and early melanomagenesis. Expression of the VDR has been shown to be reduced in highly pigmented melanomas and in the surrounding skin, and these tumours have a poorer prognosis [38]. Similarly, the expression of VDR is significantly higher in melanomas of less advanced, compared to more advanced, stages [39]. These genetic findings lend weight to the importance of local levels of 1,25(OH)_2_D_3_, which are, in turn, dependent on levels of the substrate, local or circulating 25(OH)D.

We observed a much lower prevalence of vitamin D serum 25(OH)D levels <50 nmol/L (36%) in patients with melanoma than the 78% [16] and 74% [17] reported by others, probably because of the more tropical study setting. The previous studies were undertaken at latitudes above 49°North [16, 18], whereas our study was conducted at 27°South, and over a twelve month period. Nevertheless, the observed prevalence of levels <50 nmol/L was higher than that recently reported for Queensland in the Australian Health Survey (11%) [20]. Lower 25(OH)D levels in people with melanoma compared to healthy controls have been previously reported [17], although here the different vitamin D assays used in the two studies may also have contributed to the differences observed.

A major strength of this study is the collection of blood samples within days of diagnosis, before potential changes in sun-exposure patterns occurred. The “real-time” serum collection also allowed us to adjust for season in all multivariable analyses. This is particularly important, as 25(OH)D levels vary according to the season of the blood draw [40, 41]. The measurement of total 25(OH)D concentrations in one laboratory ensured standardization of the assay technique. Further, we were able to comprehensively adjust for potential confounders. The high recruitment rate (98%) and the sample size (*N* = 100) which represents approximately 9% of the melanomas diagnosed annually in the Brisbane area [42], make it more likely that our sample was representative of the population eligible to take part in this study. The sample is also representative with respect to the subtypes and anatomic sites of melanoma seen in Queensland. For example, the most common melanoma subtype seen in this study, superficial spreading melanoma (54%), was also the most commonly reported invasive melanoma subtype to the Queensland Cancer Registry in the period 1982–2008 (55%) [43]. In the same period, the trunk (34%) was the most common site of melanomas diagnosed in Queensland [43]. In our study 37% of melanomas were located on the trunk.

In addition to these strengths, there were some limitations to our study. Firstly, this was a cross-sectional study specifically examining vitamin D status in relation to Breslow thickness, at the time of diagnosis of melanoma. Although Breslow thickness is considered to be a good marker of prognosis, we do not have data on the outcomes for these patients with melanoma and information on the pTNM characteristics, stage and melanization of the tumours was not available. Further, we cannot infer that the associations shown are cause and effect, although the genetic studies lend weight to an important role of local 1,25(OH)_2_D_3_ levels. However, we cannot infer that vitamin D supplementation pre-diagnosis will be associated with lower Breslow thickness at diagnosis, or that post-diagnosis vitamin D supplementation will affect prognosis. Randomized controlled trials of vitamin D supplementation are required to address these questions.

There is, however, value in examining the potential public health implications of our findings. Only 9% of our participants were taking a vitamin D supplement. This means that the 25(OH)D levels of the participants largely reflect their recent sun exposure (since dietary intake is very low in the Australian population) [44]. If there was a causal association of a similar magnitude to that shown here, then ensuring that all members of the population achieved vitamin D levels of 50 nmol/L or greater through the same mechanism as used by these participants, would potentially result in approximately 18% of diagnosed melanomas having a Breslow thickness of <0.75 mm, rather than ≥0.75 mm, with potential survival gains.

This study provides support for an association between vitamin D deficiency (<50 nmol/L) at the time of diagnosis and thicker melanoma at presentation (Breslow thickness ≥0.75 mm). The study was specifically examining the relationship between vitamin D status and several histopathological makers of melanoma prognosis, and not the risk of developing a melanoma. We found no evidence to support that higher levels of past sun exposure, in this low latitude, high ambient UVR setting, were associated with markers of more favorable prognosis. Brief (casual) periods of sun exposure to a large surface area (avoiding sunburn) are most efficient for vitamin D production. Maintaining a vitamin D level of ≥50 nmol/L is advisable as our results indicate that vitamin D deficiency is associated with thicker melanomas, which have, separately, been shown to have poorer prognosis. The benefits of sun exposure to achieve vitamin D “sufficiency” must be weighed against the risks of skin cancer development [44].
